# Evaluating the effectiveness of the SHEL model in reducing occupational exposure risks among pre-hospital emergency personnel: a retrospective cohort study (2018–2024)

**DOI:** 10.3389/fpubh.2026.1777003

**Published:** 2026-03-31

**Authors:** Yu Li, Xiaojuan Hou, Mingyi Qin

**Affiliations:** 1 Department of Emergency, The Fourth Hospital of Hebei Medical University, Shijiazhuang, Hebei, China; 2 Department of Endocrinology, The Fourth Hospital of Hebei Medical University, Shijiazhuang, Hebei, China; 3 Department of Nursing, The Fourth Hospital of Hebei Medical University, Shijiazhuang, Hebei, China

**Keywords:** emergency medical services, occupational exposure, pre-hospital emergency personnel, SHEL model, training effectiveness

## Abstract

**Background:**

Pre-hospital emergency personnel face significant occupational exposure risks, yet traditional training approaches often prove inadequate. This study evaluated the effectiveness of SHEL (Software, Hardware, Environment, Liveware) model-based training versus standard operating procedure training (SOPT) in reducing occupational exposure incidents.

**Methods:**

This retrospective cohort study included pre-hospital emergency personnel from an emergency center between January 2018 and June 2024. Participants were divided into SOPT and SHEL groups based on the training received. Knowledge, Attitude, and Practice (KAP) scores and safety behavior compliance were assessed before and 6 months after training. Occupational exposure incidents and root causes were analyzed throughout the one-year follow-up. Psychological status was evaluated using the Nursing Stress Scale and Maslach Burnout Inventory before and 6 months post-training.

**Results:**

The analysis included 129 and 114 staff in the SOPT and SHEL groups, respectively. Six months post-training, the SHEL group demonstrated significantly higher KAP scores and safety behavior compliance (86.41 vs. 79.83, *p* < 0.001). The SHEL group had substantially fewer individuals experiencing occupational exposure (10.53% vs. 27.91%, *p* < 0.001) and fewer total incidents (12.28% vs. 32.56%, p < 0.001), particularly biological exposures. Root cause analysis attributed a lower proportion of incidents to software factors in the SHEL group (14.29% vs. 47.62%, *p* = 0.027). The SHEL group also showed significantly lower stress (66.43 vs. 71.36, *p* = 0.004) and burnout scores (depersonalization: 37.09 vs. 35.61, *p* = 0.001).

**Conclusion:**

Training based on the SHEL model is more effective than traditional SOPT in enhancing occupational safety, reducing exposure incidents, and improving the psychological well-being of pre-hospital emergency personnel. These findings support the need for more integrated training approaches in emergency medical services.

## 1 Introduction

Occupational exposure is defined as accidental contact with blood, body fluids, sharps, chemicals, or physical hazards (1). Occupational exposure for healthcare workers, particularly pre-hospital emergency personnel, refers to contact with blood, body fluids, or other potentially infectious materials during patient care, posing risks of transmitting bloodborne pathogens such as HBV (Hepatitis B Virus), HCV (Hepatitis C Virus), and HIV (Human Immunodeficiency Virus) (2, 3). Clinical consequences can range from acute anxiety and prophylactic treatment to seroconversion and chronic infection (4). While standard precautions and post-exposure protocols are the cornerstone of prevention, the dynamic and unpredictable nature of emergency field settings often impacts their consistent application (5). Current clinical management primarily focuses on post-event prevention and treatment, which, although crucial, represents a reactive rather than proactive strategy. The limitations of traditional training are becoming increasingly apparent, as it often emphasizes theoretical knowledge rather than integrated system performance in complex environments (6, 7). Therefore, this study aims to evaluate the effectiveness of comprehensive interventions based on the SHEL (Software, Hardware, Environment, Liveware) model in enhancing occupational exposure protection for pre-hospital emergency personnel, surpassing traditional training paradigms.

The SHEL model, originally developed for aviation safety, is now increasingly applied in healthcare institutions to analyze the causes of human error and integrate organizational, environmental, and technical controls (8, 9). The SHEL model is an analytical framework that conceptualizes the interfaces between software, hardware, environment, and liveware, providing a structured approach for understanding and improving system safety (10). In healthcare, incidents are rarely caused by a single factor but are usually the result of interactions between these factors. Previous studies in various clinical settings have shown that a systems-oriented approach addresses not only individual compliance issues but also procedural deficiencies, equipment design, and environmental constraints, making it more effective in reducing errors and adverse events (11). Similar benefits have been reported for intravenous medication safety in emergency departments, where medication error rates decreased after SHEL implementation (12). The potential link between poorly integrated SHEL systems and increased occupational exposure risk lies in the cumulative effect of latent failures, such as unclear protocols, safety device malfunctions, cluttered workspaces, and communication breakdowns, which create conditions for accidents, especially in high-risk pre-hospital care (13).

This retrospective cohort study aims to determine whether SHEL model training is superior to standard operating procedure (SOP) training in reducing occupational exposure incident rates among pre-hospital emergency personnel. Its innovation lies in applying a holistic system safety model to occupational protection within the challenging context of emergency medical services and using a set of multidimensional outcome measures to assess its impact. These findings are expected to provide evidence for developing more effective and integrated training strategies, ultimately enhancing the occupational safety and overall well-being of frontline emergency responders.

## 2 Methods

### 2.1 Study design

This study is a retrospective cohort study aimed at evaluating the protective effects of enhanced training based on the SHEL model on occupational exposure among pre-hospital emergency personnel. The study included pre-hospital emergency personnel working at the emergency center from January 2018, to June 2024, and divided them into two groups based on the training they actually received, the Standard Operating Procedure Training (SOPT) group and the SHEL Training group. All personnel completed training according to the institution’s unified arrangements during their respective periods. The grouping was entirely based on existing training history data and was not influenced by researchers, nor did it reflect the personal choices of the study subjects.

Data were collected by reviewing electronic personnel records, occupational exposure reporting systems, and training logs. Follow-up data for 1 year after group allocation were gathered for all included personnel, and the incidence rates of occupational exposure events in the emergency field were compared between the two groups.

The study protocol has been reviewed and approved by the Institutional Ethics Committee (Approval Number: 2023KS118). Given that the study involves anonymized retrospective data analysis, informed consent was waived.

### 2.2 Inclusion and exclusion criteria

This study includes pre-hospital emergency personnel who have been working in pre-hospital emergency settings from 2018 to 2024, requiring them to possess a nationally recognized healthcare practice qualification certificate and have worked continuously in the emergency team for more than 6 months; they must also have complete records of occupational exposure events during the study period. Complete records specifically refer to those in the institution’s occupational exposure reporting system that must include the following core elements for verification: the date and location of the exposure incident, the mode and source of exposure, immediate on-site handling measures, related subsequent medical evaluations and follow-up results, and the final incident classification. For records in the system that lack the above key information or contain logical contradictions that prevent verification, the corresponding individuals will be excluded.

Exclusion criteria include: individuals without a valid practice qualification or those who resigned, transferred positions, or accumulated more than 30 days of leave during the study period; cases lacking complete occupational exposure records or with incomplete or unverifiable record information; pre-hospital emergency personnel with a history of severe immunodeficiency diseases, currently receiving immunosuppressive treatment, or diagnosed with infectious diseases; participants involved in other interventional occupational safety studies or those who have received other non-SHEL model protection programs; temporary staff such as interns and visiting scholars.

### 2.3 Training methods

In the SOPT group of this study, pre-hospital emergency personnel received standardized training based on the national “Occupational Protection Technical Specifications.” The training content included the concept of occupational exposure, common hazard factors, standard protective measures (such as correctly wearing protective clothing, gloves, masks, and protective goggles), and post-exposure reporting and handling procedures. The training schedule included quarterly centralized lectures lasting 2 h; quarterly on-site drills using simulated emergency scenarios for practical operations, also lasting 2 h; and an annual comprehensive assessment consisting of written and skills tests, with those scoring below 80% required to undergo retraining. All training sessions were organized by the Hospital Infection Control Department. The lecturers and instructors were all dedicated personnel from this department with over 5 years of practical experience in hospital infection management and holding provincial-level or higher infection control professional training certification. Training records were uniformly archived. The design of this program refers to the “theoretical teaching combined with scenario simulation” training model, which has been proven to significantly enhance healthcare professionals’ knowledge levels regarding occupational exposure management (14).

The SHEL team conducted systematic and contextualized comprehensive interventions in addition to regular training, focusing on the four elements: software (S), hardware (H), environment (E), and liveware (L). Monthly online module learning (30 min per session) covers various elements, emphasizing their interconnectivity. In the “Software” module, a dynamic protection process decision tool based on risk assessment was introduced, and several key operating procedures were updated, including sharps handling protocols (such as prohibiting recapping needles, standards for carrying sharps containers on-site), dual-check processes for high-risk operations, and the time points and pathways for reporting after exposure. In the “hardware” module, it focuses on the principles and correct operation of new safety devices. In the “environment” module, it explains standardized zoning and infection control strategies for different sites. In the “liveware” module, training addresses the dual dimensions of the Liveware element as conceptualized in the Edwards & Hawkins framework: the individual level and the interpersonal level. At the individual level, psychological safety training guides participants to identify cognitive biases under high pressure and encourages proactive reporting of safety hazards or operational concerns without fear of blame through case studies. At the interpersonal level, team communication training integrates standardized communication tools in emergency handovers and practices ensuring clear instruction delivery and confirmation in high-noise, high-pressure scenarios. Each module is followed by targeted platform tests to reinforce learning outcomes.

Quarterly on-site scenario drills (2 h per session) are conducted in highly realistic emergency settings. The core objective is to integrate the four elements in practice. Drills assess not only individual skills but also whether teams can correctly select and operate protective equipment based on real-time risk assessments and maintain effective team communication and role coordination in simulated chaotic environments. Post-drill debriefing sessions (1 h per session) are critical improvement phases where a multidisciplinary team reviews recent real or simulated exposure cases following the SHEL framework for root cause analysis. Exposures may result from unclear process guidelines, equipment failures, poor lighting affecting operations, and untimely team communication. Based on these analyses, targeted joint improvement measures such as revising procedures, upgrading equipment, optimizing vehicle layouts, or arranging communication skills retraining are developed and updated. The entire training cycle’s planning, execution, and iterative optimization are coordinated by a dedicated cross-departmental safety management team. The monthly online learning and quarterly comprehensive drills designed for the SHEL group are based on the SHEL model’s emphasis on the continuous identification and integration of system elements. This regular, cyclical training model helps transform systemic safety thinking into stable team practice capabilities (15).

At the end of the study period, this training model was incorporated into the center’s routine occupational safety training system. SHEL training is implemented on an annual cycle, with all personnel required to complete a full round of module learning and refresher training every 2 years. The SOPT group maintains its original annual assessment and retraining mechanism. This arrangement aims to sustain long-term safety behavior through periodic reinforcement.

### 2.4 Data collection

The data come from the emergency center’s historical archives. KAP questionnaires and the Maslach Burnout Inventory are part of regular monitoring, with personnel assessed at hiring and every 6 months. Data were extracted from records between January 2018 and June 2024, with management procedures, questionnaires, and scoring criteria remaining consistent due to standardized protocols.

#### 2.4.1 Baseline data

In this retrospective study, baseline data were sourced from the hospital’s standardized electronic record system used for routine management. All demographic and occupational information of personnel was obtained from the formal personnel file system maintained by the Human Resources Department. Vaccination histories were verified through the immunization registry system managed by the Infection Control Department. Occupational exposure histories over the past year were obtained by reviewing records from the specialized database of mandatory reported and audited occupational exposure incidents.

In the present study, task type was categorized based on risk level into high-risk scenarios and medium-low risk scenarios. High-risk scenarios included trauma/traffic accidents, cardiac arrest/CPR, obstetric emergencies, and poisoning/overdose; medium-low risk scenarios included general internal medicine/surgical emergencies, chronic disease transfers/referrals, and minor conditions/no intervention needed.

#### 2.4.2 Training outcomes

Before the training and 6 months after the training began, all participants were surveyed using a self-designed “Pre-hospital Emergency Personnel Occupational Protection Knowledge, Attitude, and Practice (KAP) Questionnaire.” The questionnaire consists of three dimensions, knowledge dimension mainly examines the understanding of standard precaution principles, protective equipment selection, post-exposure emergency handling procedures, and other basic theories and regulations, with a total of 15 questions and a full score of 30 points. The attitude dimension assesses the perception of occupational exposure risks, recognition of the value of protection, and the willingness to perform safe operations, with a total of 10 questions and a full score of 40 points. The behavior intention dimension measures the subjective willingness and tendency to take protective measures proactively, execute safety procedures, and communicate safely with the team in complex and changing emergency scenes, with a total of 10 questions and a full score of 30 points. The total score of the questionnaire is 100 points, with higher scores indicating stronger occupational safety knowledge, more positive safety attitudes, and stronger subjective intentions to adopt safety behaviors among emergency personnel. The development of this questionnaire followed a standard procedure to ensure its validity and reliability. First, based on an extensive literature review and structured interviews with five experts in pre-hospital emergency care and infection control, an initial item pool was formed to ensure content validity. Subsequently, exploratory factor analysis was conducted on the pre-survey data to ultimately confirm the three-dimensional structure of the questionnaire, verifying its construct validity. The final version of the questionnaire demonstrated good reliability, with an overall Cronbach’s alpha coefficient of 0.794.

Quality control personnel who received unified training used structured on-site observation methods. These observers were senior nurses with over 5 years of experience in pre-hospital emergency care or infection control. Before data collection, they all underwent specialized training for the scales used in this study, including theoretical explanations, video case calibration, and on-site simulated scoring exercises to ensure consistency in scoring criteria. Before and after the training, three patient transport tasks were randomly selected for each emergency responder without knowledge of the team’s tasks. An assessment scale containing 10 core safety behaviors was used for recording. This scale rated each behavior on a three-level scoring system: “full compliance 2 points,” “partial compliance 1 point,” and “non-compliance 0 points,” which was ultimately converted into an overall compliance score on a percentage basis. Higher scores indicated better compliance. Additionally, the proportion of emergency personnel members achieving the “full compliance” standard in key safety practices was calculated.

#### 2.4.3 Occupational exposure incidence and characteristics

The occupational exposure-related data were all derived from a standardized occupational exposure reporting and monitoring system established and mandatorily enforced by the emergency center. The institution requires that all occupational exposure incidents must be reported, and a multi-layer review process from incident reporting to final verification has been established to ensure the reliability of the data. Core outcome measures, including the number of exposed individuals, total incidents, severe incidents, and workdays lost due to these incidents, were extracted from this system.

After an occupational exposure incident occurs, relevant personnel must fill out a detailed “Occupational Exposure Incident Report Form” within a specified time frame through the system, providing detailed descriptions of the exposure process, potential pathogens, severity of injury, and detailed records of initial handling. Subsequently, the report must first undergo preliminary factual review by the department head and then professional verification and follow-up by dedicated personnel from the hospital infection management department. The latter verifies the post-exposure handling procedures, related medical records, and cross-checks personnel attendance records to confirm the accuracy of workdays lost. The system undergoes regular internal audits to ensure the completeness and standardization of reporting and handling.

Biological exposure is defined as contact with patient blood, body fluids, or injuries from contaminated sharps (16). Chemical exposure includes accidental contact with chemical substances such as disinfectants and chemotherapy drugs. Physical exposure encompasses mechanical injuries, radiation, etc. Each incident was independently classified by a researcher according to preset criteria to ensure consistency in categorization.

When an exposure event occurs, an investigation team composed of the head nurse of the department and infection control specialists conducts a review by verifying the on-site situation, interviewing relevant personnel, examining operation records and equipment status, and determining root causes based on the preset SHEL classification standards. Incidents due to failure to strictly enforce dual-person verification, unfamiliarity with emergency response plans, and omissions in standard precaution steps are categorized as software factors. Incidents due to damaged gloves, improper use of sharps containers, and safety device failures are categorized as hardware factors. Incidents due to insufficient lighting, space limitations inside vehicles, and interference from family members are categorized as environmental factors. Incidents caused by hurried operations leading to self-injury, communication conflicts, and teamwork errors are categorized as human factors.

#### 2.4.4 Psychological status and well-being outcomes

Before the training and 6 months after the training began, the Nursing Stressor Scale was used to assess the occupational stress levels of emergency personnel (17). This scale contains a total of 34 items, using a 5-level scoring method, with a total score range of 0–136 points. Higher scores indicate greater perceived occupational stress among emergency personnel. The Cronbach’s alpha coefficient of this scale is 0.770.

The status of occupational burnout was assessed using the Maslach Burnout Inventory, which includes three dimensions: emotional exhaustion, depersonalization, and personal accomplishment (18). Higher scores in emotional exhaustion indicate more severe depletion of emotional resources; higher scores in depersonalization indicate a stronger negative and indifferent attitude towards work; the personal accomplishment dimension is reverse-scored, with higher scores indicating stronger professional achievement and lower levels of burnout. The Cronbach’s alpha coefficient of this scale is 0.93.

### 2.5 Statistical analysis

All statistical analyses were performed using SPSS software (Version 26.0; IBM Corp., Armonk, NY, USA). Continuous variables were tested for normality using the Shapiro–Wilk test. Data conforming to a normal distribution are presented as mean ± standard deviation and were compared using the independent samples t-test. Categorical data are expressed as frequency (percentage) and were analyzed using the Chi-square test. A *p*-value < 0.05 was considered statistically significant.

## 3 Results

### 3.1 Baseline characteristics of the study participants

The baseline demographic and work-related characteristics were compared between the SOPT group and the SHEL group (Table 1). There were no significant differences in age, gender distribution, marital status, working hours, years of service, job titles, education levels, dispatch frequencies, task type, occupational exposure history, and vaccination rates between the two groups (all *p*-values were greater than 0.05). The two groups showed a high degree of consistency in these characteristics, providing a solid foundation for subsequent analyses.

**Table 1:** Comparison of baseline demographics and work-related characteristics.

Indicator	SOPT group (*N* = 129)	SHEL group (*n* = 114)	*t*/*χ*^2^	*p*
Age	33.58 ± 5.17	33.04 ± 4.84	0.836	0.404
Gender (Female/Male)	85 (65.89%) / 44 (34.11%)	77 (67.54%) / 37 (32.46%)	0.074	0.785
Marital status			0.250	0.617
Married	74 (57.36%)	69 (60.53%)		
Unmarried or divorced or widowed	55 (42.64%)	45 (39.47%)		
Weekly working hours	42.54 ± 5.27	42.28 ± 5.15	0.392	0.696
Years of service	6.29 ± 2.34	6.53 ± 2.59	0.766	0.445
Job title			0.057	0.811
Junior healthcare staff	71 (55.04%)	61 (53.51%)		
Intermediate healthcare staff	58 (44.96%)	53 (46.49%)		
Highest education level			0.249	0.883
Diploma	57 (44.19%)	54 (47.37%)		
Bachelor’s degree	61 (47.29%)	51 (44.74%)		
Master’s degree	11 (8.53%)	9 (7.89%)		
Number of dispatches per year	298.05 ± 41.63	302.07 ± 39.82	0.766	0.444
Task type	*N* = 38,449	*N* = 34,436		
High-risk scenario	24,223 (63.00%)	21,539 (62.55%)	1.591	0.207
Medium-low risk scenario	14,226 (37.00%)	12,897 (37.45%)		
Occupational exposure history in the past year	18 (13.95%)	15 (13.16%)	0.033	0.857
HBV vaccination rate	105 (81.40%)	98 (85.96%)	0.919	0.338
Influenza vaccination rate	90 (69.77%)	84 (73.68%)	0.457	0.499

### 3.2 Training outcomes

**Figure 1:**
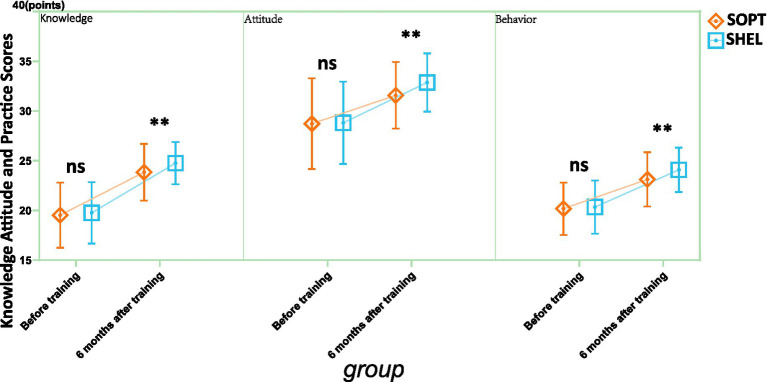
Comparison of knowledge, attitude, and practice scores before and after training. SOPT, Standard operating procedure training; SHEL, Software, hardware, environment, liveware; ns, no significant; **p < 0.01.

In the comparison of KAP scores, there were no significant differences in any of the indicators, including knowledge, attitude, and behavior scores, between the two groups before the training (all *p*-values were greater than 0.05; Figure 1). Six months after the training, the knowledge scores in the SHEL group were significantly higher than those in the SOPT group (*p* = 0.005), indicating that the SHEL group performed better in knowledge acquisition. Similarly, the attitude scores (*p* = 0.002) and behavior scores (*p* = 0.003) were also significantly higher in the SHEL group compared to the SOPT group, suggesting that the SHEL group showed more significant improvements in attitude and behavior. These results indicate that the SHEL group demonstrated stronger effects in knowledge acquisition, attitude change, and behavior improvement.

There was no significant difference in safety behavior compliance scores between the two groups before the training (*p* = 0.682; Table 2). Six months after the training, the safety behavior compliance scores in the SHEL group were significantly higher than those in the SOPT group (*p* < 0.001), indicating that the SHELG (SHEL-based Occupational Protection Training Group) performed better in safety behavior compliance after training. In specific key behaviors, the compliance rate for immediate hand hygiene after procedures was significantly higher in the SHELG group compared to the SOPTG group (*p* = 0.001). Similarly, the compliance rate for correct sharps disposal (*p* = 0.008) and the compliance rate for wearing appropriate PPE throughout (*p* = 0.019) were also significantly higher in the SHELG group compared to the SOPTG group.

**Table 2:** Comparison of safety behavior compliance and specific safe practice rates.

Indicator	SOPT group (*N* = 129)	SHEL group (*n* = 114)	*t*/*χ*^2^	*p*
Compliance before training	72.84 ± 8.16	73.26 ± 7.83	0.410	0.682
Compliance at 6 months	79.83 ± 6.57	86.41 ± 4.22	9.397	< 0.001
Key behavior				
Immediate hand hygiene after procedures	84 (65.12%)	95 (83.33%)	10.352	0.001
Proper disposal of sharps	90 (69.77%)	96 (84.21%)	7.031	0.008
Wearing appropriate PPE throughout	87 (67.44%)	92 (80.70%)	5.485	0.019

PPE, personal protective equipment.

### 3.3 Occupational exposure incidence and characteristics

Comparison of occupational exposure incidence and severity between the two groups showed significant differences in the number of affected individuals and the number of events (Table 3). The number of individuals experiencing occupational exposure was significantly higher in the SOPT group compared to the SHEL group (*p* < 0.001). The number of reported occupational exposure events was also significantly higher in the SOPT group than in the SHEL group (*p* < 0.001). There was no significant difference in the number of severe events between the two groups (*p* = 1.000), and no severe events were reported in the SHEL group. The number of workdays lost due to occupational exposure-related illnesses or injuries also showed a significant difference between the two groups (*p* = 0.013), with workday losses being more common in the SOPT group due to occupational exposure.

**Table 3:** Occupational exposure incidence and severity outcomes.

Indicator	SOPT group (*N* = 129)	SHEL group (*n* = 114)	χ^2^	*p*
Number of individuals affected	36 (27.91%)	12 (10.53%)	11.533	< 0.001
Number of incidents	42 (32.56%)	14 (12.28%)	14.031	< 0.001
Severe events	1 (0.78%)	0 (0.00%)	None	1.000
Workdays lost due to occupational exposure-related illness or injury			8.728	0.013
0 days	92 (71.32%)	99 (86.84%)		
1–3 days	31 (24.03%)	13 (11.40%)		
More than 3 days	6 (4.65%)	2 (1.75%)		

**Figure 2:**
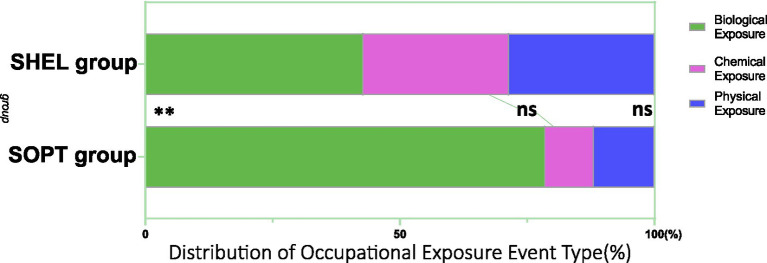
Distribution of occupational exposure event type (NS, no significant; **p < 0.01).

In the distribution of occupational exposure event types between the SOPT group and the SHEL group, the incidence of biological exposure events was significantly higher in the SOPT group compared to the SHEL group (*p* = 0.029; Figure 2). However, there were no significant differences in the proportions of chemical exposure and physical exposure events between the two groups (chemical exposure: *p* = 0.186; physical exposure: *p* = 0.294).

Analysis of the root causes of occupational exposure events based on the SHEL model showed that a significantly higher proportion of events in the SOPT group were attributed to software factors (*p* = 0.027; Table 4). There were no significant differences in the proportions of hardware, environment, and liveware factors between the two groups (hardware: *p* = 0.259; environment: *p* = 0.834; personnel: *p* = 0.855).

**Table 4:** Root cause analysis of exposure events based on the shel model.

Indicator	SOPT group (*N* = 42)	SHEL group (*n* = 14)	*χ* ^2^	*p*
Software	20 (47.62%)	2 (14.29%)	4.891	0.027
Hardware	7 (16.67%)	5 (35.71%)	1.273	0.259
Environment	6 (14.29%)	3 (21.43%)	0.044	0.834
Liveware	9 (21.43%)	4 (28.57%)	0.033	0.855

### 3.4 Psychological status and well-being outcomes

Nursing Stress Scale scores showed that there was no significant difference in stress scores between the two groups before the training (*p* = 0.971; Table 5). Six months after the training, the stress scores in the SHEL group were significantly lower than those in the SOPT group (*p* = 0.004).

**Table 5:** Nursing Stress Scale scores.

Indicator	SOPT group (*N* = 129)	SHEL group (*n* = 114)	*t*	*p*
Before training	83.85 ± 13.78	83.79 ± 12.96	0.036	0.971
6 months after training	71.36 ± 15.76	66.43 ± 10.36	2.914	0.004

MBI scores indicated that there were no significant differences in the EE, DP, and PA scores between the two groups before the training (all *p* values greater than 0.05; Table 6). Six months after the training, the EE scores in the SHEL group were significantly lower than those in the SOPT group (*p* = 0.003), indicating that the SHEL group was more effective in reducing emotional exhaustion. The DP scores were also significantly lower in the SHEL group compared to the SOPT group (*p* = 0.001), suggesting that the SHEL group performed better in reducing depersonalization tendencies among emergency personnel. Additionally, the PA scores were significantly higher in the SHEL group compared to the SOPT group (*p* = 0.017), indicating that the SHEL group was more effective in enhancing personal accomplishment among emergency personnel.

**Table 6:** Maslach Burnout inventory scores.

Indicator	SOPT group (*N* = 129)	SHEL group (*n* = 114)	*t*	*p*
Before training				
EE	23.58 ± 4.84	23.26 ± 4.31	0.547	0.585
DP	7.92 ± 1.65	7.98 ± 1.61	0.285	0.776
PA	31.72 ± 4.98	32.16 ± 4.74	0.709	0.479
6 months after training				
EE	23.75 ± 4.49	22.11 ± 3.88	3.020	0.003
DP	7.36 ± 1.59	6.72 ± 1.46	3.264	0.001
PA	35.61 ± 5.12	37.09 ± 4.33	2.408	0.017

EE, emotional exhaustion; DP, depersonalization; PA, personal accomplishment.

## 4 Discussion

This study demonstrates that, compared to traditional SOP, a systems-oriented training paradigm can produce meaningful improvements across a range of safety-related outcomes. This intervention is associated with a reduction in the frequency of occupational exposure events, a shift towards fewer software-related failures in potential pathogenic mechanisms, and measurable benefits in mental health.

The first notable phenomenon relates to the exceptional knowledge gains observed after the SHEL-based program. Participants who received SHEL training reported a deeper understanding of infection control principles, risk assessment tools, and procedural safeguards. This aligns with previous research showing that educational interventions that embed knowledge within the context of real work flows tend to result in more enduring learning (19). Studies in intensive care settings have shown that simulation-enhanced modules reflecting actual patient care scenarios retain infection prevention concepts better than purely lecture-based formats (20). The multifaceted nature of SHEL training, which addresses not only technical knowledge but also risk perception, communication skills, and decision-making abilities, may contribute to this increased efficacy (21). In contrast, traditional training often emphasizes abstract policies without embedding them in the practical operations of emergency care, which may limit its translational effectiveness (22). The SHEL cohort exhibited more positive attitudes towards occupational safety, demonstrating higher relevance and personal accountability, which aligns with the behavioral science literature emphasizing the integration of safety attitudes with organizational values. When staff view protective measures as essential for task success rather than burdensome, they are more likely to internalize and advocate for these practices (23). Real-time software components such as team communication drills, psychological safety discussions, and leadership engagement further reinforce this attitude shift.

The exceptional safety behavior compliance observed in the SHEL group, particularly in key practices such as immediate hand hygiene, correct sharps disposal, and consistent use of PPE, represents a critical finding with direct implications for exposure prevention. These behavioral improvements may stem from the SHEL model’s systematic approach to addressing both individual and situational factors that influence safe practices. While traditional training typically focuses primarily on individual capabilities and compliance, the SHEL model also targets hardware reliability, environmental design, and team dynamics, which significantly affect the feasibility and consistency of safety behaviors in actual operations (24). Studies in other clinical settings have similarly shown that interventions targeting multiple system elements result in more substantial and sustainable improvements in safety behaviors compared to those focusing on single factors (25).

The most clinically significant finding of this study is the reduction in occupational exposure incident rates in the SHEL group, highlighting the practical relevance of the intervention. Compared to the SOPT group, the reduction in exposure incidents in the SHEL group was not only reflected in the number but also in the nature of the incidents. The shift away from software-related root causes such as protocol non-compliance or inadequate risk assessment indicates that the SHEL curriculum successfully mitigated potential procedural weaknesses (26). The decrease in exposure frequency suggests that even modest improvements in software can eliminate the most common failure pathways, resulting in significant impact (27). This is particularly true for biological exposures, which are the most common and consequential exposure category in pre-hospital emergency care. The actual reduction in injuries underscores the real-world effectiveness of the SHEL-based approach in translating improved knowledge and behaviors into concrete safety outcomes (28). In one study, a disinfection supply center reported reductions in sharps injuries and contamination exposures after implementing SHEL analysis (29). Similarly, studies in emergency room settings have documented reductions in occupational exposure incidents following comprehensive safety interventions that address multiple system elements simultaneously (30). The SHEL approach effectively targeted the specific mechanisms behind these events, which often involve complex interactions between procedural compliance, equipment functionality, environmental constraints, and team communication (30).

The root cause analysis of exposure incidents based on the SHEL framework revealed interesting findings regarding the distribution of causal factors between the two groups. In the SOPT group, a higher proportion of incidents were attributed to software factors, whereas the root causes of incidents in the SHEL group exhibited a different distribution pattern, with relatively more incidents attributed to hardware and environment factors. Indicating that traditional training left significant gaps in the organizational systems supporting safe practices. This finding echoes previous research showing that software elements play a crucial role in preventing adverse events in high-risk healthcare environments (31). The lower incidence of software-related events in the SHEL group likely reflects the provision of more robust procedural infrastructure and clearer guidance through training, including standardized protection processes and risk assessment tools (32).

Improvement in mental health is a significant collateral benefit arising from the systemic intervention of the SHEL model. This study found that the SHEL group exhibited lower levels of stress and burnout after training, which not only confirms the intrinsic link between a safe environment and mental health but also more deeply reveals the pathway through which the SHEL model proactively builds psychosocial protective mechanisms through its core structure. Traditional safety training often focuses on regulating individual behavior, while the advantage of the SHEL model lies in its systems perspective, clearly defining and integrating psychological safety and team collaboration efficiency as key safety elements (33). In this study, this integration directly alleviated critical stressors leading to burnout through two core mechanisms. First, by fostering a no-blame practice culture and case reflection, it reduced employees’ situational fear of reporting errors and seeking help. This sustained sense of psychological safety decreases common emotional masking and feelings of isolation experienced during high-pressure work, thereby directly buffering against emotional exhaustion (34). Second, by embedding standardized communication tools and team emergency decision-making processes into high-fidelity drills, it provided shared thinking and action guidelines for dealing with chaotic situations. This reduces each member’s cognitive load and anxiety associated with reconstructing communication patterns and facing decisional uncertainty in emergencies, which are important factors inducing occupational alienation (35). Therefore, the promotion of mental health by the SHEL model is not an incidental byproduct of its physical safety goals but rather a result of systematically reducing unnecessary cognitive and emotional resource depletion caused by the work system itself, thus creating a more supportive and predictable work experience for Liveware. This aligns with previous research findings, which show that healthcare professionals who feel supported by strong systems, clear protocols, and effective teamwork experience lower levels of work stress and burnout, even in highly sensitive environments (36).

Despite these promising findings, several limitations of our study warrant consideration. The two training programs differ in content, frequency, and structure, with the SHEL group having higher engagement. Therefore, the improved outcomes in the SHEL group may partly be attributed to the higher training frequency rather than the specific content of the SHEL model itself. This factor makes it difficult to attribute all benefits solely to the SHEL content. As a retrospective cohort study, the grouping was entirely based on existing training records. The research team could not trace the specific decision-making process that assigned different personnel to SOPT or SHEL training, which may have involved batch replacements of training programs, differences in hiring times, or other institutional arrangements. This is an inherent limitation of the retrospective design. Despite efforts to ensure baseline equivalence between groups, there may still be potential confounding factors. The retrospective design carries a risk of reporting bias. SHEL training promotes more active identification and reporting of occupational exposure, while the SOPT group may have lower awareness or reporting willingness due to different training content, leading to potential underreporting. If the control group underreports, their actual exposure rate may be higher, making the observed difference (lower exposure rate in the SHEL group) a conservative estimate. Although a mandatory standardized reporting system was used to mitigate this bias, it cannot be entirely excluded. Single-center implementation limits generalizability, as organizational culture, resource availability, and patient populations vary by setting. A six-month follow-up period for primary outcomes, though sufficient to detect initial training effects, is insufficient to assess long-term sustainability, a critical consideration for occupational safety initiatives. Future studies should address these limitations through multi-center randomized designs with extended follow-up periods, more nuanced assessments of implementation barriers and facilitators, and economic evaluations to determine cost-effectiveness. Investigating how specific SHEL components contribute to overall effectiveness will also help refine and optimize the intervention model.

## 5 Conclusion

This study provides strong evidence that training based on the SHEL model improves occupational exposure protection for pre-hospital emergency personnel across multiple dimensions. Improvements in knowledge, safety behaviors, exposure rates, and mental health underscore the value of addressing occupational safety through a comprehensive, systems-based perspective that acknowledges the complex interactions between software, hardware, environment, and liveware elements. These findings are significant for developing more effective and comprehensive approaches to protect frontline emergency responders working in unique challenges and high-risk environments.

## Data Availability

The original contributions presented in the study are included in the article/supplementary material, further inquiries can be directed to the corresponding author.
